# Understanding Knowledge Mobilisation between Community Champions and Parents: Evidence from a Community-Based Programme to Support Parents with Young Children

**DOI:** 10.3390/children11080901

**Published:** 2024-07-26

**Authors:** Kath Wilkinson, Vashti Berry, Jenny Lloyd, Georgina Marks, Iain Lang

**Affiliations:** 1 College of Medicine and Health, University of Exeter, St Luke’s Campus, Heavitree Road, Exeter EX1 2LU, UK; v.berry@exeter.ac.uk (V.B.); j.j.lloyd@exeter.ac.uk (J.L.); i.lang@exeter.ac.uk (I.L.); 2 Action for Children, Chestnut Family Hub, Exeter EX2 6DJ, UK; georgina.marks@actionforchildren.org.uk

**Keywords:** community support, champions, parenting, implementation, knowledge mobilisation, realist

## Abstract

Background: Community champions have been employed across various settings to disseminate evidence-based public health information. The Building Babies’ Brains programme trains champions to work with parents in communities, equipping them with child development knowledge and parental engagement strategies. We explored what makes community champions effective in distributing information to parents, including how the champion–parent relationship and champions’ personal characteristics affect information dissemination. Methods: Champions included both peers and professionals working with parents in target communities. We administered an online survey (*n* = 53) and follow-up interviews (*n* = 14) with champions, with representation from across all training cohorts. We conducted a realist-informed reflexive thematic analysis to generate themes in the data and highlight the contexts, mechanisms, and outcome patterns identified. Results: We observed 15 Context–Mechanism–Outcome configurations across five themes: information sharing opportunities, information relevance, the nature of the champion–parent relationship, interaction expectations, and champion confidence. Our programme theory for how the community champion approach works identified that peer champions focused more on building rapport, modelling behaviours, and being a trusted community resource than direct information transfer. Professional champions, in contrast, showed greater expertise and confidence in discussing parenting practices directly. For both groups, traits such as friendliness and the ability to establish a trusting relationship enhanced effectiveness. Conclusions: This research identifies the impacts of champion role, characteristics, and the champion–parent relationship on the effectiveness of knowledge mobilisation in this context, with implications for training and recruitment of champions. Those using a champion model in comparable settings should ensure that champions have the necessary knowledge, skills, and confidence to engage parents and share information effectively.

## 1. Introduction

### 1.1. Early Interventions to Improve Child Health

The experiences of a child during their early years of life are of critical importance to their cognitive, social, and emotional development [1,2]. This development depends heavily upon a sensitive and responsive relationship with their caregiver [3,4], with shared early experiences providing lifelong benefits [5,6]. There is growing evidence that parenting programmes based on Social Learning Theory [7] improve the health and well-being of parents and children [8,9]. Recent pan-European evidence suggests that these benefits do not vary by social disadvantage [10]; however, there is differential reach in the uptake of parenting programmes with poorer attendance for low-income parents; reasons for this include access (group timing or location), acceptability (social stigma, lack of knowledge or awareness), as well as personal situation (poor mental health, social anxiety). With children from these families at increased risk compared to their peers [11,12,13], those parents with the greatest potential to benefit appear to be the least likely to engage [14]. The recent Academy of Medical Sciences report regarding UK health promotion [15] focuses on the importance of effective early interventions to improve child health and well-being and calls for services and communities to work together to deliver effective services for children and families across the nation. Evidence points to the need for an alternative approach to traditional parenting support which engages parents from across the socio-economic spectrum [16]; empowering communities to support healthy, resilient, and connected populations [17] has the potential to increase parent reach and reduce the risk for widening health inequalities.

In the following sections of the Introduction, we explore how community-based approaches have been used in public health interventions and consider this approach for the provision of parenting support. We then outline the background and aims of the current study, within which we attempt to investigate the potential opportunities and challenges to an alternative approach to parenting support employing community champions.

### 1.2. Interventions Utilising Community Assets

Over the last two decades, researchers have begun to explore the mechanisms underlying health interventions using community assets [18,19,20]. One of the most common strategies employed is the use of peer volunteers/champions to spread evidence-based health information across community networks [21,22]. In the UK, we usually refer to ‘community champions’ as individuals drawn from the target community, who are not typically trained health professionals but who receive some training to do a variety of roles; these individuals usually function as peer support or bridging roles between communities and services [23]. Intervention programmes can support this process through recruiting and training ‘community health champions’ to promote health. In general, these approaches work through utilising and enhancing the skills, knowledge, and commitment of individuals, thereby building community capacity. While the focus is often on the delivery of community-based programmes, a key mechanism is the utilisation of natural, or in some cases, created, social networks to reach underserved communities [24]. It is generally agreed that peer support embodies several overlapping dimensions, commonly described as: emotional, affirmational, informational, and practical support [25]. Theories such as Social Cognitive Theory [26], the Transtheoretical Model of Health Behaviour Change [27], the Social Mobilization Approach [28], and Diffusion of Innovation Theory [29] have all been used to describe the community-based approaches to improving public health using volunteers.

The idea that people can become health leaders, motivators, and educators in their communities is not new; this approach has been used across a range of public health interventions, such as sexual health [30,31,32,33], dental health [34], domestic abuse [35], female genital mutilation [36], nutrition [37], smoking cessation [38], breastfeeding support [39], alcohol consumption [40], cancer awareness [41], mental health and well-being [42], older people’s health and fitness [43,44], wider health promotion [45], and COVID-19 response and recovery [46,47]. In their international review, South and colleagues [22] found several reviews evidencing the positive impact of lay health workers on the health and well-being of UK service users [48,49] and increasing knowledge and awareness of health issues within the populations and groups with whom they work [50,51,52,53]. There is also evidence of improved access to and uptake of services that utilise volunteers in low-income groups [50,54], for example: mammography educational interventions [55], oral health promotion [34], and through empowering communities and building capacity to design and develop local services [56]. There are also promising findings of behaviour change beyond improving reach, such as positive lifestyle changes and improved self-management of conditions (e.g., diabetes [53,57]), as well as some evidence of improved health outcomes [58].

Studies have started to elucidate the successful components of community champion programmes, including how best to identify those in positions of social influence within a community and the personal qualities considered necessary for maximum effect [46,59]. To date, community-based approaches to health promotion have received significantly less attention within the literature than more traditional public health prevention programmes [21].

Community champions have the potential to reach families who need support but who are not meeting the threshold for targeted support from professionals, or who are unknown to local services. Research shows that parents from low-income families are less likely to believe in or seek out help for parenting than those with higher incomes, but when they do, they are most likely to turn to family and friends for support rather than professionals [60,61]. Further, Barlow and Stewart-Brown [62] found that one of the key factors identified as beneficial to parenting programmes was not the content of the programme but the support received from other attending parents. This mutual identification has also been highlighted by others [63]. It follows that providing peer champions with the necessary knowledge and skills will benefit parents, particularly those from lower-income and other harder-to-reach communities.

### 1.3. The Current Study

Our study focuses on understanding the dissemination of information by community champions who have completed the Building Babies’ Brains training programme run by the charity Action for Children. The programme was delivered out of Devon County Council’s Family Hubs, which constitute a single place for children and families to access support (e.g., baby groups, parenting classes, counselling, and financial advice). Running since 2018, the programme aims to harness relationships that already exist within community groups, by identifying, training, and supporting community champions to disseminate accessible, evidence-based parenting and child development information as part of their everyday interactions with parents. The programme aims to reduce health inequalities by targeting disadvantaged communities and aspires to build community capacity by training multiple champions within a single community to mobilise knowledge. One of the theoretical underpinnings of the programme is that successful knowledge transfer between individuals in society relies heavily upon social capital; that is, “the networks of relationships among people who live and work in a particular society, enabling that society to function effectively” [64] (Diffusion of Innovation Theory [29]). The programme also works on the assumption that people can both influence and are influenced by their environment, recognizing the effects of cognitive processes (conceptions, judgements, motivations) on an individual’s behaviour (Social Cognitive Theory [26]). This study seeks to build on these theories to further understand how community champions can support knowledge mobilisation within communities of parents.

Our study investigates the use of both peers and professionals as community champions, supporting the idea that disseminating evidence-based information from multiple sources within a community is more likely to be effective in achieving its aims [31]. These champions are individuals working within the community who encounter parents in a professional capacity (e.g., as childcare providers). Whilst there is a suite of research exploring the use of champions within professional contexts to drive behaviour change within organisations, implement interventions, etc., research around the use of community champions has, to date, tended to focus solely on peers acting as the champions.

In our research, we aimed to investigate the following questions:

- What are the differing contexts within which champions interact with parents in the community, what impact do they have on the sharing of evidence-based information, and why?
- What impact does the nature of the champion–parent relationship have on how much, and via what methods, evidence-based information is shared with parents?
- What are the individual traits and characteristics of effective community champions working with parents of young children (of which there has been limited research to date (e.g., [65]))?

In addition to answering these questions, we aimed to further develop and refine a programme theory for sharing evidence-based information with parents to mobilise knowledge within a community setting.

## 2. Materials and Methods

### 2.1. Participant Selection

We invited all individuals who had attended the Action for Children’s Building Babies’ Brains (BBB) training to participate in our study. Devon’s Family Hubs cover a population in the southwest of England comprising around 814,000 people, spread out across both rural and urban areas. The sample population was comprised of 151 individuals who were trained across 16 different groups between September 2018 and May 2022. These groups spanned both rural and urban communities across different areas of Devon. Individuals were contacted by email via Action for Children and were sent an information sheet and link to the anonymous online survey. A post was also placed on the private BBB champion social media page including a link to an online information sheet. At the end of the survey, participants were provided with the option to leave their contact information to take part in a follow-up interview. We sought to interview participants until we reached sufficient ‘conceptual depth’ in our understanding and ability to theorise from the data [66].

### 2.2. Data Collection

We obtained University ethical approval for our research prior to inviting champions to participate. An information sheet including contact details for further information about the study was emailed to participants with the invitation to take part and posted alongside the social media post advertising the research. Further information as well as a consent form were included in the online survey, and there was a requirement for this to be agreed to before proceeding to the survey questions. Participants involved in the interviews were also required to complete an additional consent form prior to taking part, and all participants were informed of their right to withdraw participation at any point during the research.

We included a survey and interviews in our research design both to gather information from a range of champions whilst also enabling the collection of more in-depth insight from a smaller group of champions. The appropriateness of the design in answering the research questions, collection of sufficient research data, recruitment of participants, and research practicalities were all considered in the design of this research.

The survey questions and interview topic guide (see Appendix A and Appendix B) were developed in consultation with Action for Children staff involved in the development and delivery of the BBB training. The survey included both closed and open questions, and the semi-structured interviews enabled further exploration of champions’ experiences and views. Specifically, we explored both the frequency and ways in which champions interacted with parents in their community and the strategies they used to pass on information, champions’ confidence in conversing with parents and sharing information, and whether these things changed over time. We also investigated champions’ views about the most important personal characteristics for champions to possess, the importance of the champion–parent relationship in passing on information, champions’ strategies for building relationships with parents, and their views about parent receptivity to information shared. Finally, we asked champions to share examples of when they perceived a positive change for a parent following the sharing of information, and their thoughts about the community champion approach and its sustainability more generally.

The survey was completed between May and July 2022, and the interviews between June and July 2022. Survey completion took approximately 10–15 min, and interviews were approximately 30 min long (range = 21–34 min). Interviews took place either over the phone or virtually, via Zoom (version 6.0.3, San Jose, CA, USA).

All the interviews were conducted by the first author, who has significant experience in leading research interviews. They were digitally recorded (with participant permission), transcribed verbatim, and anonymised. All consented participants provided complete interview data; there were no withdrawals.

Champions were offered shopping vouchers as an incentive to take part, with £5 for survey completion and a further £10 for the interview. There was also the option of donating the voucher to a local community group supporting children and families.

### 2.3. Data Analysis

We used a reflexive thematic analysis process [67] to analyse the interview transcripts along with the open-ended survey questions. This involved initial familiarisation and immersion with the dataset followed by the development of a list of potential codes to capture the important features of the data relevant to answering our research questions. Two members of the research team were involved in this process. We then developed a coding framework to manage, organise, and reduce the data based on our underlying theories and assumptions (deductive) and to capture unexpected codes (inductive) (see Appendix C). Once the coding framework was agreed upon, the interview transcripts along with relevant descriptive data and survey responses were coded by the interviewer, and a selection of them were also double-coded by a member of the research team. We then examined the codes to begin to develop themes within the data. Discussions took place amongst the team to ensure that all relevant information had been captured. Using framework analysis, we separated the coded data according to how the champion encountered parents in the community:

- Professionals: champions encountering parents in a paid position, e.g., working in schools, childcare settings, local councils, charities, NHS, community development and public libraries, or foster care assessments;
- Group leaders: champions running community groups for babies/toddlers and parents (some paid, some volunteers);
- Peers: champions who encounter parents as other parents, qualified peer supporters (e.g., breastfeeding), or charity volunteers supporting families directly.

To investigate the impact of differing contexts of the champion–parent interaction, the champion–parent relationship, and the effects of champion’s personal characteristics on the sharing of evidence-based information, we adopted a realist-informed approach [68] for generating hypotheses within the next stage of analysis. Realist evaluation recognises the influence of socio-historical context on how people use and respond to programmes: things do not simply “work” or “not work” but may work some of the time, for certain people, in certain situations; “what works, for whom, under what circumstances, and how?” [69]. We identified and set out the contexts and mechanisms by which community champions achieved their aims and expressed these in terms of Context–Mechanism–Outcome configurations (CMOcs; [70]). Together, these CMOcs provide us with a more detailed programme theory of generative causation about when and how community champions pass on evidence-based information to parents. The CMO framework helps to reflect on the relationships between context, mechanisms, and outcomes in the programme through developing hypotheses about which mechanisms are likely to ‘fire’ in which contexts and the outcomes associated with these.

## 3. Results

### 3.1. Participant Characteristics

Fifty-three champions (35% of the sample population) responded to the survey, with respondents from across all 16 training groups. This varied from between one and seven people per training group and included champions from 11 different geographic areas in Devon, as well as four area-non-specific training groups delivered virtually (see Table 1).

Community champions who completed the survey held various roles in the community where they encountered parents (see Table 2). Half of the champions worked with parents in a professional capacity (28; 53%); almost a quarter (11; 22%) of these were involved in foster care and associated parenting assessments. A third of champions worked at or ran community groups for parents and children (17/53; 32%), and the remainder encountered parents as peers (8; 15%).

Twenty-five champions initially expressed an interest in taking part in the interview, and 14 (26%) completed a consent form. Given that it was practically achievable, we decided to interview all 14 champions who consented rather than to determine the point at which we reached sufficient conceptual depth to answer our research questions (as we had planned). Eight interviews took place over the telephone, and six virtually (via Zoom). These champions covered 11 different training courses spanning 2018 to 2022 (see Table 1) and held a variety of roles within the community where they encountered parents. The sample of champions who took part in the interviews consisted of 13 females and one male; six professionals, five group leads, and three peers.

### 3.2. Themes

During our first stage of analysis, we coded the survey and interview data and developed five themes to explain the emerging findings. We then progressed onto a realist-informed analysis of the data where we developed CMOcs under each theme to refine our programme theory of how the community champion approach to sharing information with parents works in practice, why, for whom, and in what contexts. Below we describe the CMOcs and themes, with supporting quotations from the research.

**Theme 1. Information sharing opportunities**

Champions reported varied levels of opportunity to interact with and pass on evidence-based information to parents in their communities. This often depended on the nature in which champions encountered parents (i.e., as professionals, group leads, or peers). Peer and group lead champions were most likely to meet and converse with the same parents on a regular basis, and as a result reported being able to develop positive, trusting relationships over time.

*‘I think being open, flexible, willing to just wait and slow down until there is the right moment … there isn’t a huge hurry as soon as you meet someone (to pass on information). Maybe we have the luxury of time whereas others wouldn’t … just being able to watch first and I guess feeling confident in dealing with whatever comes as a result of that conversation.’*

*(Participant 2)* 

Where regular contact between the champion and parent was lacking, this was seen as a barrier to developing quality relationships and sharing evidence-based information with parents.

*‘With childminding … I like the doorstep handovers because I think it’s more settling for the children to come in having said ‘goodbye’ at the door, but sometimes it is hard to talk to parents because you don’t get much time.’*

*(Participant 7)* 

Champions who had pre-existing relationships with parents and who interacted regularly with the same parents were able to share information in a natural, non-direct way through their ongoing conversations, sharing information as and when appropriate over time.

*‘You start talking to them and quite often you find that people … with the family friendly cafes … they’re in on a particular day, so you make sure that you go in on said day. You always ask how they are and then eventually you start a bit more chatting.’ *

*(Participant 4)* 

**Theme 2. Information relevance**

Regular interactions between champions and the same parents in the community enabled champions to become more understanding of each parent’s personal situation, allowing them to select information to share that was most relevant. The tailoring of information to ensure maximum relevance to parents was also mediated by how knowledgeable and confident the champion felt about the information they were sharing.

*‘I think the most important thing is to be more aware of the person rather than the message you’re trying to pass on, otherwise I think you run the risk of putting people off by trying to force something onto them, and as much as you know it’s incredibly helpful and important, you have to make sure that your audience is receptive to it, and that it’s right for them.’*

*(Participant 9)* 

*‘It could be difficult or inappropriate to pass it on to people whose situation we don’t know.’*

*(Anonymous)* 

The regularity of contact champions had with the same parents also impacted *how* information was shared; for example, seeing the same parents often made it possible to gradually impart information over time. Drip-feeding information in this way ensured that parents were not overwhelmed by information and provided champions with the opportunity to get to know parents and gauge the relevance of information prior to sharing.

*‘The first week of baby massage I give them lots of factual information so I don’t tend to say much that week but get a good gauge of the parents and where they are, how confident they are with their babies, what dynamics might be going on and stuff.’*

*(Participant 2)* 

**Theme 3. The nature of the champion–parent relationship**

Almost all champions in our research agreed that having a pre-existing, positive, and trusting relationship between a champion and a parent was important to pass on information effectively, though not all thought that this was essential. Champions who encountered parents as non-professionals believed that the quality of the peer relationship was key to champions feeling confident about sharing information with parents, as well as determining how information would be received by the parents.

*‘I was running the group for nearly four years, so some of them had been there with their first child and second child … they were definitely easier to chat to … I might be passing knowledge over without even realizing I was doing it … it was more natural.’*

(Participant 5)

*‘If you do see that same parent again … you know you can open up the conversation again, and you feel more confident to do so because you’ve bonded before.’*

*(Participant 13)* 

Peer and group lead champions often took time to develop trusting relationships with parents they encountered regularly and positioned themselves as equals through sharing personal information about their own lives and parenting journeys, becoming trusted acquaintances with parents over time.

*‘It’s important for them to know that you’re on the same page as them and have that experience too, because having small children is difficult and exhausting! I think knowing that the person that you’re talking to has children and understands that is helpful. You can be the perfect parent but sometimes your child is just going to have a meltdown in the middle of a supermarket.’*

*(Participant 4)* 

Certain champion characteristics were also highlighted as being important for relationship-building, such that parents are most likely to develop positive relationships with champions who are friendly and approachable, as parents felt at ease in their company. Being sensitive to parental pressures (e.g., sleep deprivation, the cost of childcare, pressures to be a ‘good’ parent, managing difficult behaviour), and demonstrating shared experience and understanding were also highlighted as important for champions interacting with parents in the community.

*‘I think it’s really important that parents feel comfortable with that person (the champion), so they can talk, and that person listens to what they’re saying. And I really do feel that it makes a difference. I think it’s important that that person is approachable and welcoming and friendly, and it’s the same every week.’ *

*(Participant 7)* 

Further, champions perceived parents to be more receptive to information being shared by someone they knew and trusted compared to a stranger.

*‘Parents get a lot of (unwanted) advice which can be confusing. There has to be an element of trust to believe what you are saying.’*

*(Anonymous)* 

**Theme 4. Interaction expectations**

Champions who encountered parents in a professional capacity reported finding it easy to share information with parents due to their experience conversing with parents and because the nature of the champion–parent interaction was such that parents were expecting information and advice to be given.

*‘In work I’m already in that client-support worker role, so they expect me to be giving them some advice and having extra knowledge and stuff … and they’re ready to take it.’ *

*(Participant 8)* 

Conversely, where the relationship between the champion and the parent was non-professional, champions reported sometimes finding it difficult to steer the conversation in order to share evidence-based information; this was mostly apparent where champions were not overly confident in conversing with parents, in the information they were sharing, or they did not see the same parents regularly and therefore did not have the pre-existing relationships upon which to build and tailor their conversations.

*‘I think sometimes I struggle to find a window with people. I don’t always know if that is me, or whether someone is quite unapproachable and doesn’t want to know stuff. It’s always superficial and you don’t really get past that or have permission to, and it always tends to be the kind of people who need to hear it the most.’ *

*(Participant 2)* 

Champions encountering parents in a non-professional capacity often used modelling and reinforcing positive parenting behaviour as ways of sharing information in a more naturalistic way.

*‘I’ve been trying to attune with the Mum and validate her feelings. It’s tricky, I find it much more difficult working with peer parents … When the anxious parent comes in, that’s where it’s really difficult … all I can do is just be the same with my daughter, let her explore the world and not tell her off, and then maybe she’ll see that nothing bad is happening and let her daughter do the same.’*

*(Participant 8)* 

*‘It’s about creating those situations … everything positive and reassuring, even if they’re not asking for it. Also, that can help me to bond with that mother as well.’*

*(Participant 13)* 

**Theme 5. Champion confidence**

All champions involved in the research agreed that being confident talking to parents was more important for sharing evidence-based information than the position a champion held in the community. Champions also believed that parents would be more receptive to information coming from a champion who displays confidence in what they are saying.

*‘Somebody who has confidence and feels quite passionately about those key messages, and I’d say good communication skills. It’s more about the individual than the experience or role.’ *

*(Participant 14)* 

Where champions reported interacting with parents regularly, they described being more confident in sharing information, which meant they did so more often. The champions involved in our research who interacted with parents in a professional capacity reported feeling most confident to share information following champion training. Indeed, as a group, the professionals also noted that the training was more about gaining knowledge than gaining confidence to converse with parents. The ease with which professional champions were able to pass on information to parents may also have been helped by the expectation that this was a normal part of the professional–parent interaction, as previously discussed.

*‘I’ve been working with parents and talking to parents and broaching tricky subjects quite a long time … so it doesn’t faze me.’*

*(Participant 7)* 

Conversely, some non-professional champions reported feeling unsure how to deal with questions or challenges from parents during interactions, resulting in them feeling less confident overall in sharing evidence-based information. This was particularly prevalent where the champion–parent relationship was not well established. In these situations, champions reported focusing on sharing information that was most briefly and easily explained, such as the importance of back-and-forth parent–child interactions, as opposed to child development neuroscience to help understand a child’s behaviour.

*‘I guess one of your fears is that people will challenge you and you won’t know the answer … that’s a little bit the case with everything in life … there’s limitations on how much you know.’ *

*(Participant 11)* 

*‘A lot of people I may be talking to at the groups that I’m supporting, I don’t know them, I don’t know their situation. I don’t want to be a trigger. I don’t want to make it look like I’m passing judgement either. If you say the wrong thing and that person is not going to be back for a week … you don’t want that sort of responsibility.’ *

*(Participant 13)* 

Almost all champions reported that their confidence improved with time, due to practice, positive reactions from parents, further training and reflection, or a change in personal circumstance (such as becoming a parent themselves).

*‘I feel more confident … I’ve built up ways to approach people about key topics … if it comes to sleeping or tantrums or being fussy with food, you have a little speech in your head that you can go to. The more you talk about the brain, the more examples you can bring in as well, from people that you’ve helped.’*

*(Participant 6)* 

**Figure 1:**
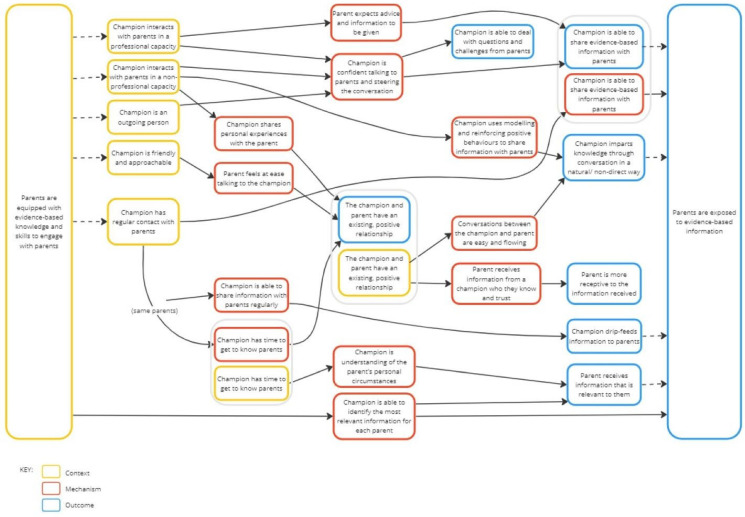
Visual representation of the Context–Mechanism–Outcome configurations.

Table 3 lists the 15 CMOcs, and Figure 1 provides a visual representation of the CMOcs to demonstrate the overlapping and ‘ripple effect’ nature of some of these [73] (‘ripple effect’: the idea that the outcome of one CMOc can become the context of another).

## 4. Discussion

The aim of our research was to investigate the factors that influenced the sharing of evidence-based information about parenting and child development by community champions. We explored the nature of the interaction and relationship between champions and parents and how this impacted champions’ views of the frequency of sharing information, how information was shared, their perceptions of parental receptivity, and why. We also investigated champion characteristics and confidence, and if, how, and why these impacted the above. We used our findings to develop our programme theory for how and why a community champion approach to sharing evidence-based information could work in early-years’ parenting support, with the potential of engaging parents from across the socio-economic spectrum.

Our findings corroborate those from our previous research with community champions, highlighting the importance of creating a ‘receptive context’ for passing on information to parents [74]. This appeared of most relevance to champions working with parents in a non-professional capacity, who underlined the importance of a pre-existing, positive, and trusting relationship with parents in facilitating information sharing. Previous research by Metz and colleagues [75] also emphasizes the importance of relationships in sharing information, stating that high-quality relationships among implementation stakeholders was a—if not the—critical factor for achieving implementation results. In their subsequent paper, Metz and colleagues [76] outline both the relational strategies for trust-building in relationship development (quality, reciprocity, and mutuality of interactions) as well as the technical strategies (demonstration of knowledge, reliability, and competency). For professional champions, the nature of the champion–parent relationship and the expectation of the interaction meant that less emphasis was placed on the importance of taking time to get to know parents and building a relationship. These champions were generally more experienced in conversing with parents and more confident in sharing information. Our findings suggest that community champion approaches should include professionals where they can; this aligns with social prescribing approaches where the community connection pathway has been formalised in health services [77].

For champions interacting with the same parents regularly, this enabled development of the champion–parent relationship over time and, in turn, allowed champions to introduce evidence-based information gently and gradually to parents that was most relevant to them in a natural, conversational way. Indeed, regular conversations have been widely reported in the literature as an important feature of effective information sharing [30,31]. Existing research also suggests that the effect of sharing information is greatest when target population members hear repeated messages, many times and in different words, from multiple champions in their own circles of friends [30,31]. The greatest challenges for non-professionals identified in our research were having limited opportunities to pass on information to parents and a lack of confidence to steer conversations to share the intended information. Indeed, corroborating previous research, in interventions where the context has not primarily been a setting for social interaction, the passing on of information has been found to be less successful [30,31,32,33]. Benefitting from a larger sample than we used in our study, future research would profit from investigating the impact of the geographical context (the area characteristics and local population) on the effectiveness of community champion approaches, considering that areas with greater cohesion and stronger existing social networks may report greater effectiveness due to increased opportunities to share information with parents.

Our research also investigated the traits and characteristics of champions considered important for successful knowledge mobilisation within communities. In addition to champions having the confidence to converse with parents and share information, several personal characteristics including being friendly and approachable were highlighted by champions as important for parent receptivity, perhaps especially so for peer champions given our finding that peers rely more heavily on developing new relationships with parents than professionals to share information. This supports previous research identifying personal qualities in champions such as being approachable, empathetic, open, motivated, credible, and persuasive [78,79] as key for making community champion programmes successful. Being sensitive to the strains put upon parents and demonstrating experience and understanding were also highlighted as central for champions working with parents in the community. This finding has implications for champion recruitment, with the most effective champions having regular contact with parents and the confidence and personal characteristics necessary to build relationships and share information. There are also implications for champion training, including ensuring that key points of evidence-based information to share with parents are easy to understand and communicate, and that champions are trained in strategies to engage parents as well as the evidence-based content. A further point worth investigating is how champion agreement or belief in the evidence-based information to be shared impacts both their confidence to share and their likelihood of sharing information with parents. While the current study confirmed that champions believed in the potential impact of the champion approach, we did not specifically investigate this question, which could also have implications for champion training.

Some argue that peer education holds the potential for spreading forms of pedagogy which are relatively free of the authoritative relationships typically associated with professionals’ teaching [80]. Our research identified several benefits of peer champions for the sharing of evidence-based information to parents in the community. These include peers being able to get to know parents over time, drip-feeding information relevant to individuals, modelling positive parenting behaviours, and providing a trusted and approachable source of information and support. Models of peer education hypothesise that peers make effective health educators because they are more credible messengers of health information, building on existing social networks, and modelling good practice [81]. A basic premise of Social Cognitive Theory (a cognitive formulation of Social Learning Theory; [7]) is that people learn not only through their own experiences, but also by observing the actions of others and the results of those actions, and through reinforcement. The latter can also help with parental self-efficacy, which is a person’s confidence in their ability to act and achieve change. Moreover, existing research holds that the consistency and credibility of champions as role models is central to the effective diffusion of information [59]. Peer support for ‘hardly reached’ parents in high-income communities has been used for a multitude of health concerns, and the review by Kaks and Malgvist [82] indicates that this has generally been well received with a variety of quantifiable improvements, despite variations in intervention design. Peer support has also been found to be a valuable social support resource for parents of children with neurodevelopmental disorders [83], disabling conditions [84,85], and children with complex needs [86], but further high-quality studies are needed to ascertain the true potential benefits and harms of these interventions. The review of peer education and peer counselling reviews by Topping [87] found positive effectiveness of interventions in terms of both knowledge and behaviour changes but warns that the approach requires careful consideration and monitoring for potential to be realised.

It is important to acknowledge that there are other key features of successful community champion programmes that have an impact on the effectiveness of information sharing that were not investigated here, such as: participation in training [46], participatory approaches [54,88], volunteering qualifications [89], ongoing support for champions—both from programme leads [56,63] and peers [88], and having a supportive infrastructure, long-term investment, and meaningful community engagement [46]. Future research would also benefit from investigating what is happening in practice within communities, as well as the sustainability of this approach over time, given the ethical considerations of failing to pay adequate attention to sustaining programmes mobilising community efforts [90].

### 4.1. Strengths and Limitations

Our research develops an initial programme theory for how community champions can be used to share evidence-based information with parents in communities to support parent–child relationships and long-term child development outcomes, in which circumstances, and for who this strategy might be most effective. It identifies interesting differences between how and why professional and non-professional champions operate to share information and the impact this may have on the effectiveness of the champion model. Our research also highlights some key benefits of employing peer champions and what considerations need to be made when recruiting, training, and supporting champions to share information and mobilise knowledge within communities.

We employed primarily qualitative methods for this research in order to gather in-depth data from participants to answer our research questions and followed the COREQ qualitative research reporting checklist [91] to ensure that all relevant information was included in the paper. We are confident that our research meets quality standards for qualitative research as indicated in the sections above, including the eight criteria outlined by Tracy [92]. However, it is important to note that this type of research has its disadvantages too, including researcher influence and subjective interpretation of data. Involving multiple research project members in both project and instrument design as well as the interpretation and coding of the data helped to mitigate these factors.

It is important to note that our research focuses on *if* and *how* the evidence-based information was shared with parents by community champions, not the impact the information had on the parents in the community. Furthermore, because we did not speak with parents directly, all reports of parent ‘receptiveness’ to information shared by champions, the relevance of the information shared, and the quality of the champion–parent relationship was reported by champions; parent perspectives may differ. Future research would benefit from gathering this information from parents as well as champions, and identifying the impact the shared information has on parental knowledge, beliefs, and behaviour, and ultimately the child–parent relationship and child health and well-being outcomes.

Given that we do not have information about the characteristics of the champions who chose not to take part in our study, we cannot be sure of how representative our participants are of the champion population. Our categorisation of community champions as professional and non-professional may also have oversimplified reality, given that there may be stark differences between how different types of professionals (e.g., health versus education) interact with the parents they encounter, and that many professionals can additionally act as peers in different contexts. Future research would benefit from including a larger sample of champions to allow separation of professional roles and exploration of potential differences in champion–parent interactions. As highlighted in the discussion section above, a larger sample would also allow for exploration of any impact of the geographical context (the area characteristics and local population) on the effectiveness of community champion approaches.

### 4.2. Practice and Policy Implications

The findings from our research have implications for both policy and practice. For example:

- Building community capacity and investing in community champion programmes have the potential to successfully enable the sharing of information with parents from across the socio-economic spectrum, reaching families who often fail to engage with traditional parenting support programmes.
- Programmes should aim to recruit and train both professionals and non-professionals who interact with parents to act as community champions and mobilise evidence-based information.
- Champion recruitment should consider that champions who interact with the same parents regularly are more likely to be able to develop trusting relationships with parents; a factor which has been identified as key to successful information sharing, through providing a foundation from which to gradually pass on relevant information.
- Hearing the same messages from multiple sources has been shown to increase the chance of parents taking information on board, making a case for community-wide strategies and the targeted training of multiple champions within the same locality for maximum impact.
- Recruitment should also take into consideration the personal traits and characteristics of champions, as those who are friendly, approachable, and have greater confidence conversing with parents are most likely to pass on information effectively.
- Professional and non-professional champions often share information with parents in different ways and have different requirements when it comes to training. This should be taken into account in programme design and implementation.
- Champion training should include strategies for engaging parents as well as key points of evidence-based information to share with parents.

## 5. Conclusions

Our research explores how community champions have the potential to provide valuable information and support to parents across communities. Non-professionals can provide a helpful addition to professionals (champions or otherwise), benefiting from being part of the target population, having the time to get to know parents and develop positive, trusting relationships, drip-feeding relevant information, and modelling and reinforcing positive parenting behaviour. It is important to note, however, that while most professionals often do not have the time to get to know parents in the same way that many peers may do, it does not mean that their relationship is less positive or that the information shared with parents is less relevant. Indeed, the fact that they are professionals changes the dynamics of the relationship they have with parents, and they may bring greater experience in identifying parental needs and assessing the relevance of information to share with them.

These findings will be of interest to those designing community-delivered public health interventions for parents of young children, with implications for champion selection considering regularity of champion–parent contact, as well as champion personal characteristics including approachability, credibility, and confidence. There are also implications for implementation and sustainability with respect to champion training and ongoing support, given that the main challenges for non-professional champions were found to be around confidence and strategies to engage parents and share information; effective champion programmes cannot be accomplished without providing ongoing opportunities for champions to practice, discuss, receive support, and resolve difficulties they encounter when acting in these roles [31,37,59]. Examples of effective programmes describe how champions are not taught just to convey educational information but about the *how* and the conversational approach to delivering that information; creating more positive attitudes and recommending practical ways to enact behaviour change [30,31,32,33]. Furthermore, in terms of champion training models more generally, it is important to note that research has found that hearing the same information on numerous occasions from numerous individuals increases the likelihood of it being taken on board.

## Figures and Tables

**Table 1:** Participants by geographic area.

Area of Devon (Date of Training; Number of Champions Trained)		Area Type [71] (Approx. Population [72])	Number of Champions in Survey (*n* = 53)	Number of Champions in Interviews (*n* = 15)
Site 1	(7 November 2021)	Urban town (31,000)	1	1
Site 2	(10 May 2019)	Rural town (5000)	2	1
Site 3	(10 September 2018)	Rural town (8000)	3	-
Site 4	(3 March 2022)	Mixed rural and urban district (151,000)	2	1
Site 5	(12 February 2019)	Urban city (131,000)	1	-
Site 6	(10 August 2021)	Urban town (12,000)	3	-
Site 7	(11 November 2021)	Urban town (12,000)	6	3
Site 8	(8 November 2018)	Urban town (27,000)	3	3
	(9 May 2022)		3	
Site 9	(6 October 2020)	Urban town (13,000)	3	-
Site 10	(9 February 2020)	Urban town (20,000)	4	-
Site 11	(14 September 2019)	Rural town (6000)	7	1
Virtual	(11 January 2021)	Mixed rural and urban areas across Devon	4	1
	(10 March 2021)		2	1
	(10 April 2021)		4	1
	(11 June 2021)		5	2

**Table 2:** Champion role in the community.

Role	Number (%) of Champions in Survey	Number of Champions in Interviews	Overarching Role Category
Runs groups with parents and children	17 (32%)	5	Group Lead
Works in an early childcare setting	10 (19%)	1	Professional
Works with parents and children in other capacities (e.g., via schools, local Councils, charities, the NHS, community development, public libraries)	12 (23%)	4	Professional
Involved in foster care assessments	6 (11%)	1	Professional
Peer supporter	3 (6%)	2	Peer
Volunteers with families	1 (2%)	1	Peer
Other	4 (8%)	-	Peer
	**53**	**14**	

**Table 3:** Context–Mechanism–Outcome configurations.

	**Theme 1: Information sharing opportunities**
1	Where the position a champion holds in the community means that they encounter parents regularly (C), they will be able to share evidence-based information about parenting and child development (M), exposing the parents they encounter to this information (O).
2	Where the champion is knowledgeable about evidence-based information around parenting and child development (C) and they encounter the same parents on a regular basis (C), they will be able to talk to parents regularly (MRes), meaning that they are able to share information with parents gradually over time (O).
	**Theme 2: Information relevance**
3	Where the champion gets to know parents in the community over time (C), they are more aware of and understanding of the parent’s personal situations (M) and can choose information to share that is most relevant to each parent (O).
4	Where training provides adequate opportunity for the champion to acquire the necessary knowledge and skills to share evidence-based information with parents (C), they are able to understand and identify what information is most relevant to individual parents (M), and parents are more likely to receive information that is relevant and useful to them (O).
	**Theme 3: The nature of the champion–parent relationship**
5	Where the position a champion holds in the community means that they encounter the same parents on a regular basis (C), they will have time to chat and get to know each other (M) and will be able to develop a positive, trusting relationship as a result (O).
6	Where the champion is friendly and approachable (C), the parent feels at ease talking to them (M), which means the champion is able to get to know the parent and develop a positive, trusting relationship with them (O).
7	Where the champion and parent have an existing, positive relationship (C), they know and trust each other (M), and the parent will be more receptive to information shared by the champion (O).
8	Where the champion and parent have an existing, positive relationship (C), conversations are easy and flowing (M), allowing the champion to pass on information in a natural, non-direct way (O).
9	Where the champion interacts with parents in a non-professional capacity (C), they are able to share information about their own parenting journey as peers (M), developing a trust and connection between the parent and champion (O).
	**Theme 4: Interaction expectations**
10	Where the champion interacts with the parent in a professional capacity (C), the parent expects information and advice to be given (M), so the champion is more able to share evidence-based information (O).
11	Where the champion interacts with the parent in a non-professional capacity (C), they need to be confident steering conversations (M), so that evidence-based information can be shared with the parent (O).
12	Where the champion interacts with the parent in a non-professional capacity where there is no expectation that information and advice will be given (C), they use alternative ways to share information with the parent such as modelling and reinforcing positive parenting behaviours (M), enabling the sharing of evidence-based information in a more natural, non-direct way (O).
	**Theme 5: Champion confidence**
13	Where the champion interacts with the parent in a professional capacity (C), the champion is experienced and confident talking to parents (M) and so feels able to deal with questions or challenges from the parent about the evidence-based information that they are sharing (O).
14	Where the champion is an outgoing person (C), they are confident approaching people and initiating conversations (M), so they are more likely to interact with parents they encounter and share information during their conversations (O).
15	Where the champion interacts with parents regularly (C), they are confident talking to parents because of their experience (M), and so are able to share evidence-based information with parents during conversations (O).

## Data Availability

The original contributions presented in the study are included in the article, further inquiries can be directed to the corresponding author.

## Appendix A. Online Survey for Community Champions of Building Babies’ Brains

You’ve been invited to take part in this survey because you have attended the Building Babies Brains training with Action for Children (previously known as Community Thrive). We’d like to ask you some questions about your experience of being a Community Champion, and to learn a bit more about how this works in practice. We are interested to know how your experience may have changed over time, and the things that make it easier or more difficult for Champions to pass on messages to parents in the community.

The survey is anonymous, and your responses will be reported as such. If you are willing to take part in a subsequent interview to discuss your answers in more detail, we will ask you to leave your name and contact information at the end of the survey—this is completely optional. Contact information will not be downloaded with survey responses to maintain anonymity. We are very grateful for any participation you give to help us to learn more about how best to support champions to help the families in their communities.

The survey will take approximately 15 min to complete.

**Consent to take part in the research study** *(Please tick each box)*

1. I confirm that I have read the information sheet dated 11 May 2022 (version 3.0) for the above project. I have had the opportunity to consider the information, ask questions and have had these answered satisfactorily.
2. I understand I am free to withdraw from the survey up until the point of submitting my answers.
3. I understand that should I withdraw participation from the interview after the data has been analysed, it may not be possible to remove anonymized data from the research report.
4. I understand that all data will be collected, stored and processed in compliance with the Data Protection Act 1998.
5. I understand that data collected during the study may be looked at by members of the research team from the University of Exeter, where it is relevant to my taking part in this research. It may also be accessed by individuals from the University of Exeter and regulatory authorities for audit purposes. I give permission for these individuals to have access to my information.
6. I understand that I will not be able to be personally identified from any data or quotes used in dissemination of the results of this research.
7. I understand that the anonymised data collected in the study may be used to support other research in the future, and may be shared with other researchers.
8. I agree to take part in the above study.

**About you**

- Please can you describe the ways in which you come into contact with parents in the community? *(open text)*
- Has this changed since you attended the training? If so, please describe your role at the time of the training. *(open text)*
- Do you participate in any other ‘champion’-type roles in the community? *(Yes/no)*
  ○ If so, please describe this briefly
- Do you undertake any other kind of voluntary work in the community? *(Yes/No)*
  ○ If so, please describe this briefly
- Have you previously taken part in a research interview about your role as a community champion following your training with Action for Children

**The training**

- Please select the course that you attended. *(list of dates and locations provided)*
- How did you come to take part in the training? *(Voluntary/nominated by workplace/other (describe))*
- Why did you decide to volunteer as a Community Champion? *(open text)*
- How confident did you feel to pass on messages to parents in your community after attending the training? *(Very confident/Confident/Somewhat confident/Not very confident/Not at all confident)*
  ○ Has this confidence reduced, increased or stayed the same over time? *(reduced/increased/stayed the same)*
  Why?

**Your experience of Pass It On**

As a reminder, Pass It On (PIO) refers to the way in which Champions ‘pass on’ information to parents in the community. This could happen in a variety of ways for various snippets of information relevant to parents and their children.

- In the few months after the training, roughly how many parents did you pass messages on to in your community? *(A lot (more than 20 parents), Quite a few (between 10 and 20), Some (between 6 and 10), A few (between 1 and 5), None (Unfortunately I did not find any opportunities to ‘pass it on’))*
- Did the opportunity to ‘pass it on’ happen more or less than you thought it would? *(More/Less)*
  ○ Why do you think this was? *(open text)*
- How important do you think the relationship a Champion has with a parent is in the ‘pass it on’ model? *(Very important (the relationship is essential in how the message is received)/Important/Somewhat important/Not very important/ Not at all important (it is all about the message))*
  ○ Please explain your choice. *(open text)*
- Please could you provide an example of information that you passed on to parents? *(open text)*

**Sustainability**

If you completed your training more than six months ago, please answer the following questions:

- More recently, how often have you ‘passed it on’ to parents in your community? *(Very often (more than twice a week), Often (once a week), Occasionally (a couple of times a month), Rarely (once a month or less), Never (Unfortunately I did not find any opportunities to ‘pass it on’))*
  ○ If you have ‘passed it on’ less than you were, what is the reason for this? *(My role in the community has changed/I have not had the opportunities to ‘pass it on’/I have forgotten much of the training and the key messages/I am not sure that ‘pass it on’ really makes much difference to parents/other)*
- Has the way in which you pass on messages changed as time has moved on? Please explain. *(open text)*
- Is there anything that would be helpful for Champions to support them to keep passing on messages some time after attending training? *(open text)*
- Do you think that the BBB model of training champions in the community is a sustainable approach to supporting parents to change their behaviours? *(yes/No/not sure)*
  ○ Please explain *(open text)*

**Final thoughts**

- Do you think that passing it on works? Do you think that you are having, or that you had, an impact on/changing parent behaviours? (Please explain) *(open text)*
- Do you think there is anything that could increase the impact that champions have on parents in their community? *(open text)*

**Next steps**

As part of this research we are also conducting interviews with community champions. We are keen to speak with champions who are still active as well as those who are not. If you could spare 30 min for a telephone or online conversation to elaborate on some of your above responses, we would be extremely grateful.

To take part, or for more information, please enter your details here:

Name: _______________

Email: _______________

Many thanks for taking the time to complete this survey.

Please note that by submitting this survey you are agreeing for your anonymised responses to be included in the research.

## Appendix B. Topic Guide: Community Champions of Building Babies’ Brains

*You’ve been invited to take part in an interview because you have attended the Building Babies Brains training with Action for Children (previously known as Community Thrive). We’d like to ask you some questions about your experience of being a Community Champion. Are you happy to be interviewed and for our conversation to be recorded?*

**Motivations for becoming a Community Champion**

1. Could you start by telling me a little bit about how you got involved in the community thrive training?

**Training**

2. When did you complete the training?
  *Prompts: Have you attended any follow-up sessions or engaged with the Facebook group since the training finished? If so, how often? How recently?*
3. Are you in the same role now as you were when you attended the training, relating to your proximity to parents?
  *Prompts: If not, does your new role enable as much/more/less opportunity to pass on messages to parents? Has the dynamic changed in any way?*
4. How prepared/confident did you feel to pass on messages and to be a champion after the training?
  *Prompts: What do you feel are the key reasons for you feeling (un)prepared/(un)confident?*

**Pass It On—experience, barriers and facilitators**

5. Since the training, have you found opportunities to ‘pass it on’?
6. Can you describe a situation where you’ve passed these messages on?
7. What is your approach to passing it on?
  *Prompts: How do you create/take those opportunities? Do you revisit that person after passing it on?*
8. When you’ve not been able to pass on these messages, what has stopped you from ‘passing it on’?
  *Prompts: What are the challenges? What is it about passing it on that is most challenging (the message, the setting, the person etc)?*
9. Has the frequency or way in which you pass on messages changed as time has moved on?

**Role of relationships in PIO**

10. What do you think are the most important characteristics of a successful community champion?
  *Prompts: Is there anything specific about their personality? Their role in the community? Their personal or professional experience?*
11. How do you think the relationship a champion has with a parent impacts on the way the message is received/taken on board?
  *Prompts: How well do you think you have to know a parent to pass on messages to them? Does this differ depending on the nature of the messages you are passing on?*
12. How can you tell if a parent is going to be open to messages—that is, hearing about evidence or strategies for parenting from a community champion?
13. Is there anything you do to create/develop relationships with parents to support PIO?
  *Prompts: Is this different for each parent?*

**Impact and sustainability**

14. Do you think that passing it on works? Do you think that you are having some impact on/changing parent behaviours?
15. How do you think this model of champions passing on messages to parents could be sustained over time?
  *Prompts: Is there any support that champions need?*
16. Do you have any other comments you would like to add?
17. Thank you for your time today. Please could I just confirm that you are happy for us to use the information you have provided today anonymously, along with the responses from other champions, to help us in our research?

## Appendix C. Coding Framework

**Initial Themes**	**Codes**	**Description**
Champion role	Peer	Champions who encounter parents as other parents, qualified peer supporters (e.g., breastfeeding), or charity volunteers supporting families directly.
	Group lead	Champions running community groups for babies/toddlers and parents (some paid, some volunteers).
	Professional	Champions encountering parents in a paid position, e.g., working in schools, childcare settings, local councils, charities, NHS, community development and public libraries, or foster care assessments.
Information sharing opportunities	Regular interactions with the same parents	Champions regularly come into contact and can converse with the same parents.
	Irregular interactions with parents	Champions do not interact with the same parents on a regular basis.
Information sharing strategies	Parents ask champions questions directly	Parents approach champions to ask for advice or questions about child development and/or parenting strategies.
	Sharing information in a group setting	Champions share evidence-based information with parents as a group, e.g., during a ‘baby massage’ class.
	Sharing information online	Champions post on social media or respond to questions from parents in online forums.
	Modelling behaviours	Champions model positive parenting behaviours and strategies in front of parents.
	Reinforcing behaviours	Champions actively reinforce positive parenting behaviours demonstrated by parents.
Parent receptiveness	Methods of gauging parent receptiveness	Reference to specific methods used or clues looked for by champions when attempting to judge parent receptiveness for information.
	Knowing the parent helps	Parents are likely to take information on board from a champion they know well.
	Confident champion helps	Parents are likely to take information on board from a champion who is confident.
Champion–parent relationship	Views on the importance of a trusting relationship	Mention of how important the champion–parent relationship is for the sharing of information.
	Pre-existing relationship	Champions and parents have a pre-existing relationship from which to share information.
	Greater ability to assess relevance of information for parents	Champions are better able to assess the relevance of information to share with parents they know well.
	Parents are expecting information to be given	Professional champions interact with parents in settings where information is expected.
Strategies used to build relationships	Take time to get to know parents	Champions see parents regularly and get to know them over time.
	Sharing personal information	Champions share information with parents about their personal lives and parenting journey.
Champion personality/ characteristics considered important	Champion confidence	Mention of champion confidence (or lack of) in conversing with parents in the community.
	Being friendly and approachable	Champions exhibit friendly characteristics and are approachable for parents.
	Having a shared experience and understanding	Champions demonstrate that they are also parents and share experiences and understanding with other parents.
Challenges to sharing information	Difficulty steering the conversation	Champions find it difficult to steer conversations in order to share information.
	Irregular interactions with parents	Champions do not interact with parents regularly.
	Champion lacking confidence	Champions report a lack of confidence in sharing the relevant information with parents.
	Unsure how to deal with challenges from parents	Champions are unsure how to respond to parents’ questions and challenges to the information shared.
	Some information is difficult to explain	Champions report struggling to share some of the more complex child development information when interacting with parents.
Motivations to take part in training	Increasing knowledge	Champions want to learn about child development and parenting strategies.
	Gaining confidence in approaching parents	Champions want to learn strategies for engaging parents and confidence in approaching them to share information.
Impact of the approach	It works	Champions believe that the community champion approach works to support parents and children. Include why.
	It does not work/not sure	Champions do not believe or are not sure if the community champion approach works to support parents and children. Include why.
	Examples of impact	Mention of examples where champions have witnessed or been told about the impact that information sharing has had on parents and/or children.
Sustainability	Confidence over time	Mention of changes in champion confidence over time.
	Top-up sessions/events	Champions suggested top-up sessions or celebration events would be useful.
	Resources for champions	Champions would welcome resources to take away or to hand out to parents.
